# The social life of the brain: Neuroscience in society

**DOI:** 10.1177/0011392113476464

**Published:** 2013-05

**Authors:** Martyn Pickersgill

**Affiliations:** University of Edinburgh, UK

**Keywords:** Brain imaging, mental health, neuroethics, neuroscience, Imageries cérébrales, neurosciences, neuroéthique, santé mentale

## Abstract

Neuroscience is viewed by a range of actors and institutions as a powerful means of creating new knowledge about our selves and societies. This article documents the shifts in expertise and identities potentially being propelled by neuroscientific research. It details the framing and effects of neuroscience within several social domains, including education and mental health, discussing some of the intellectual and professional projects it has animated therein (such as neuroethics). The analysis attends to the cultural logics by which the brain is sometimes made salient in society; simultaneously, it points towards some of parameters of the territory within which the social life of the brain plays out. Instances of societal resistance and agnosticism are discussed, which may render problematic sociological research on neuroscience in society that assumes the universal import of neuroscientific knowledge (as either an object of celebration or critique). This article concludes with reflections on how sociotechnical novelty is produced and ascribed, and the implications of this.

## Résumé

Les neurosciences sont considérées par un grand nombre d’intervenants et d’institutions comme un moyen efficace de produire de nouvelles connaissances sur nous-mêmes et sur la société. Cet article étudie l’évolution des compétences et des identités produites par la recherche neuroscientifique. Ce texte examine quelles sont les formulations et les conséquences des neurosciences dans différents domaines sociaux, notamment dans l’éducation et la santé mentale, et analyse certains des projets intellectuels et professionnels qui y ont été menés (comme la neuroéthique). Cette étude s’intéresse aux logiques culturelles qui privilégient la place du cerveau dans la société tout en mettant en évidence quelques-uns des paramètres territoriaux dans lesquels la vie sociale du cerveau joue un rôle. Des cas de résistance de la société civile et le cas de l’agnosticisme y sont discutés à la lumière des problèmes qu’ils posent à la recherche sociologique sur les neurosciences, au sein d’une société qui assume la portée universelle des connaissances neuroscientifiques. Cette étude se termine par une réflexion sur la façon dont la nouveauté sociotechnique est produite et reconnue et sur les implications de ce processus.

## Resumen

La neurociencia es normalmente considerada por una serie de actores e instituciones como un medio poderoso para la creación de nuevo conocimiento sobre nosotros mismos y las sociedades. Este ensayo documenta los cambios en el conocimiento y en las identidades que están potencialmente siendo propulsadas por la investigación neurocientífica. En él se detallan el encuadre y los efectos de la neurociencia en varios ámbitos sociales, incluyendo a la educación y a la salud mental, discutiendo algunos de los proyectos intelectuales y profesionales que ha animado la misma (por ejemplo, la neuroética). El análisis se ocupa de las lógicas culturales por las cuales el cerebro es por veces algo construido y por veces algo destacado en la sociedad. Al mismo tiempo, apunta hacia algunos de los parámetros del territorio en el cual la vida social del cerebro se desarrolla. Se discuten los casos de resistencia social y el agnosticismo, los cuales pueden resultar en una investigación sociológica problemática sobre neurociencia en la sociedad, por asumir la importancia universal del conocimiento neurocientífico. Este artículo concluye con algunas reflexiones sobre cómo la novedad sociotécnica se produce y es atribuida, así como sus implicaciones.

## Introduction

Promissory discourse that takes neuroscience as its focus frames the study of the brain as a powerful means of providing new ways for understanding our selves and societies. Neuroscientific research, including brain imaging studies, contributes to longstanding debates concerning free will, morality and madness. In so doing, it reignites the same kinds of questions about selfhood and its physical corollaries and determinants that fired the imaginations of nineteenth- and twentieth-century novelists and filmmakers, social commentators and policy actors. Increasingly, the terms and concepts of neuroscience are evident within domains and discourses that are only loosely connected to biomedicine; a consequence of this is that existing professional practices are sometimes challenged, and new social engagements and relations can be animated.

The reasons why neuroscientific research has operated in this way and had such effects on the production, circulation and deployment of knowledge are diverse. However, the historian Fernando Vidal (2009) has emphasized especially trends in cultural discourse regarding individualism, the import of identity politics, the salience of biomedical facts and the portability of (neuro)images in enabling and sustaining diverse interests in neuroscience. Sociological attention to the social life of the brain has thus been directed by the challenges (and, some believe, opportunities) neuroscience presents to the social sciences as a consequence of its causal tropes and presumed policy implications – and has been further engendered by the kinds of rapprochements with other forms of praxis that I have pointed to above (and will characterize in more detail below).

Reflecting in particular on the impact of neuroscience on mental health, social theorist Nikolas Rose has suggested that ‘we’ have now become ‘neurochemical selves’, understanding thought, feeling and behaviour as being mediated through the brain (Rose, 2007: 188). Similarly, for Thornton, ‘the language of the brain is a powerful vocabulary through which citizens today express their anxieties, articulate their hopes and dreams, and rationalize their disappointments’ (Thornton, 2011: 150). This is regarded as being, in part, a consequence of the ‘saturation of public discourse with biological and neurological ways of thinking’ (Thornton, 2011: 112). Indeed, Pitts-Taylor speculates as to whether seeing ‘ourselves in neuronal terms may be becoming a duty of biomedical citizenship’ (Pitts-Taylor, 2010: 649) and Holmer Nadesan (2002) suggests that brain science promotes new regimes of surveillance, bolsters the power of already privileged groups and extends governmentality. Such formulations, all explicitly indebted to the work of Michel Foucault, vividly capture some of the broad shifts in identity that the meta-narratives of neuroscience seem to have entrained. However, as Thomson (2005) argued for Foucauldian analyses of psychology in society, there is a risk that these conceptualizations overstate the transformations in subjectivity that have occurred with and through neuroscience (Bröer and Heerings, in press; Choudhury et al., 2012; Pickersgill et al., 2011).

In this article I juxtapose reflections on neuroscience – developed through and informed by research continuously undertaken since 2005 under the aegis of several projects (supported by the AHRC, ESRC, Newby Trust and Wellcome Trust) on the sociology of neuroscience and mental health^1^ – with accounts and analyses in other social science literature, in order to document the social life of the brain. Through the article, I undertake an examination of the power of neuroscience, and of the shifting understandings of expertise and identity potentially being propelled by neuroscientific research.^2^ The account presented aims to be sensitive to the sociotechnical innovations that have come with (and supported) the increasing attention of researchers and wider publics to the neurological, but is at the same time mindful of the limits within which these take place. This analysis thus suggests that the very premise on which much social scientific analysis of the brain is undertaken (i.e. the pervasive biomedical and cultural import of neuroscience) needs to be more closely scrutinized, underscoring the importance of examining how novelty is produced (including the role of sociological work itself in producing the significance of ‘new’ objects of study). The article thus intersects with wider debates within (medical) sociology and science and technology studies (STS) on the role of the promissory and the co-construction of expectations and agendas between social science analysts and the actors and practices being analysed (Barben et al., 2008; Brosnan, 2011; Burchell, 2009; Calvert and Martin, 2009; Hedgecoe and Martin, 2008; Macnaghten et al., 2005; Molyneux-Hodgson and Meyer, 2009).

## What is neuroscience?

Characterizing neuroscience is somewhat akin to attempting to define the social sciences; any attempt risks reducing a diverse set of discourses and practices into a ‘manageable’ rubric that perhaps effaces as much as it reveals, and presents as unified that which is highly heterogeneous. Nevertheless, we can advance a broad definition: i.e. that neuroscience is the scientific study of the nervous system, including the brain. It is at once a conglomeration of scholarly traditions (molecular biology, biochemistry, medical physics and so on) as well as a discipline in its own right (much like gender studies, or STS). Many neuroscientists are concerned with both the structure and function of neurological matter: what are the different bits of the brain, and what role do they play in both basic and more sophisticated physiological and cognitive processes? So-called ‘lesion studies’ have played an important role in neurological research (a tradition which now sometimes come within the broad purview of ‘the neurosciences’), and today genetic and cellular approaches are employed alongside computational perspectives. Neurosurgery, pharmacological disruption of neural circuits and molecular techniques are all common features of contemporary neuroscience.

Investigators have taken as their focus matters as diverse as addiction, emotion, learning, memory, sensation, sleep and, of course, psychopathology. We can thus see that the scope of neuroscience is extremely wide-ranging, and so too is its ambition. The following extract, taken from the first page of the introductory chapter of one popular undergraduate neuroscience textbook, illustrates these aspirations:

It is human nature to be curious about how we see and hear; why some things feel good and others hurt; how we move; how we reason, learn, remember, and forget; the nature of anger and madness. These mysteries are starting to be unravelled by basic neuroscience research. (Bear et al., 2007: 4)

This short extract reveals three key points. First, neuroscience commonly takes as its subject the human itself; this contrasts with older pursuits associated with the pursuit of cerebral knowledge, such as craniometry, which were specifically underpinned by efforts to empirically validate *differences* between Europeans and other hominids (Carson, 1999). Second, in spite of this, the notion of humanity undergirding much neuroscientific research is informed by modernist ‘Western’ assumptions about the singularity of human nature. Last, human nature is understood as constituted through somatic structures and processes.

In recent years, neuroscientists have come to be concerned not solely with the neurological but with sociality. The subdiscipline of ‘social neuroscience’, for instance, examines how the brain mediates social interaction and cognition (Young, 2012). This field exemplifies the jurisdictional challenge some regard neuroscience as posing to the traditional social sciences (and the humanities) (e.g. Duster, 2006), and the ways in which it reconfigures the boundaries between taken-for-granted ontological distinctions regarding ‘biology’ and ‘society’.

Similar work articulating brain science and the study of social life has, however, also been undertaken by sociologists (Franks, 2010; TenHouten, 1997; for analysis, see Von Scheve, 2011). As Webster suggested for genomics, such scholarship means that we must confront, or at least contemplate, a situation wherein the ‘very object of social science analysis – the social – is no longer clearly defined’ (Webster, 2005: 234). Research at the interface between sociology and neuroscience continues longstanding attempts by the social sciences to emphasize the soma (Dingwall et al., 2003), as well as problematizing simplistic characterizations of these disciplines being, *a priori*, ‘opposed’ to one another. Further, the fact that rapprochements between brain and social science are deemed possible and workable reminds us of the extent to which neuroscientists themselves can incorporate quite elaborate ontological imaginaries within their experimental praxis (Pickersgill, 2009). Indeed, those working in the realm of ‘critical neuroscience’ seek explicitly to create a more ‘reflexive scientific practice’ in neuroscientific research through the incorporation of insights from the social sciences and humanities (Choudhury et al., 2009: 61).

Neuroscientists, then, are not all blithely deterministic, with dreams of reducing all of human experience to the circulation of cerebral blood and the alchemy of neurotransmitters. Yet, a research focus on neuroscience and the neurological does, in many cases, result in an assignment of ontogenic privilege to the brain in reflections on and assumptions regarding personhood. However, whilst such investigations are commonly understood as helping to enjoin new understandings of selves as brain, Fernando Vidal has cogently argued the inverse: that ‘the ideology of brainhood’, which he regards as ‘the quality or condition of being a brain’, has in fact ‘impelled neuroscientific investigation much more than it resulted from it’ (Vidal, 2009: 5). For Vidal, emerging ideas about brainhood can be traced back to the mid-eighteenth century. This insight – i.e. that claims for the transformational nature of neuroscience may themselves be problematic – provides an organizing framework for the subsequent analysis presented here.

### Attending to images

In terms of the attention of sociologists, it is neuroimaging that has formed the dominant focus of empirical and conceptual work. Technologies like PET (positron emission tomography) and fMRI (functional magnetic resonance imaging) measure, respectively, blood flow or oxygen consumption, and the results are used to make inferences about brain activity. However, just as it would be inaccurate to consider survey research as the sole methodology within the social sciences, many neuroscientists do not undertake imaging research but rather make use of a wide range of tools and methods (such as those noted above). Further, whilst imaging techniques are widely used, critique remains regarding how they are and should be employed. As anthropologist Joseph Dumit has shown, a given neuroimaging technology builds ‘assumptions into its architecture and thus can appear to confirm them, while … reinforcing them’ (Dumit, 2004: 81). Neuroscientists themselves have likewise questioned the ‘puzzlingly high correlations’ seen in the results of imaging studies, and hence problematized the significance of these for casting light on the phenomena under study (Vul et al., 2009).

Imaging is perhaps also the most visible aspect of neuroscience within wider cultural discourse, and the most ubiquitous icon of neuroscientific power today appears to be the brain scan. Often compelling, these evocative representations of cerebral matter are ciphers over which a variety of professionals and publics have come to lay their own understandings of personhood. Frequently described as ‘pictures’ of the brain, though actually representations of complex statistical data, they are produced by technologies such as PET and MRI. Such techniques are commonly understood as granting unmediated and unproblematic access to what Anne Beaulieu (2000) calls the ‘space inside the skull’. Nonetheless, these scans are produced through the work of human actors existing within what is sometimes a tense social matrix that involves a range of investigators and research participants.

Structural, as well as micro-sociological, power relations mediate the relations between scientists and their subjects. In the UK, university and NHS ethics committees may place particular limitations on the actions of investigators that govern their contact with research participants, and, in the US, a ‘complicated landscape’ of human research protection has a powerful influence on the mechanisms of research (Kulynych, 2002: 346). These ‘regimes of normativity’ (Pickersgill, 2012a) not only shape the nature of relationality in science, but the emotions experienced by participants and investigators in the process. In particular, neuroscientists are under new pressure to scrutinize decisions regarding whether to disclose incidental findings from scans (e.g. the presence of tumours). In 2005, the US National Institutes of Health co-organized a conference with Stanford University, on the ‘Detection and Disclosure of Incidental Findings in Neuroimaging Research’; since then, a plethora of literature has been published in science and ethics journals on this matter, with little sense of universal consensus emerging as a consequence. Diverse forms of responsibilization are thus occurring within neuroscience; however, precisely who is responsible for what and why are not readily resolvable questions. Consequently, the legitimacy of those institutions and individuals exercising power over research processes and participants remains ambiguous. In the next section, I discuss further some of the normative dimensions of neuroscience (as perceived by sociologists and others). Through doing so, I aim to cast light on some of the sociotechnical work involved and the negotiation of ‘novelty’ essential to it.

## Neuroscience and the normative

Science, technology and medicine are aggregates of actors, discourses and practices that exert profound influence on the functioning of societies and enactment of social relations. The diverse dimensions and forms of power structuring contemporary neuroscience, and the authority of neuroscience itself, are key sociological problematics: from the micro scale of scientists interacting with those who participate in their studies, to the macro, where we can see symbolic and economic investments in brain science in order to bolster already dominant military regimes (e.g. Board on Army Science and Technology, 2009).

The links between the armed forces and science have always been complex (Bud and Gummett, 1999), with military-funded research sometimes contributing both to enhancing the destructive potential of nations, and to the improvement of health and societal infrastructure internationally. The relationship between neuroscience and the military is a case study of this moral complexity; for instance, as Lichtman and Sanes (2006) have argued, the joint sponsoring of research into the nervous system by the British Army and the British Medical Research Council during the Second World War led to what has been regarded as pioneering work. As Moreno (2006) shows, neuroscience today commands the attention of defence agencies seeking to enhance soldiers’ capabilities (for instance, via pharmaceuticals or devices aimed at heightening cognition) and improve national security (e.g. through neurotechnological deception-detection) (see also Tennison and Moreno, 2012).

In the latter case, developments in neurologic means of lie detection simultaneously are sustained by ‘post-9/11 anxiety’ and contribute to the production of ‘models of the brain that reinforce social notions of deception, truth, and deviance’ (Littlefield, 2009: 365). Equally, expectations of pharmaceutical intervention are powered by longstanding hopes and research trajectories alongside fictional tropes which legitimate attempts to actualize them (Wolf-Meyer, 2009). As such, the sponsorship of and focus on neuroscientific research by the military can be understood as resulting from the novel import the science is taken to have, which is itself reliant on pre-existing ideas about possible futures articulated through a range of media. At the same time, the novelty of neuroscience is re-embedded within cultural discourse as a *consequence* of symbolic and material investments.

The entanglements between neuroscience and the state have been taken by some commentators to be important – not least because these may be regarded as contradicting public-facing statements by funders about the explicitly health-related focus of government sponsorship of neuroscientific research. As a result of this moral claims-making, the relationships they revolve around are deserving of greater attention by sociologists. However, the military is not the only field of practice within which the normative aspects of neuroscience are constructed and debated. A new discipline of ‘neurolaw’ is today being forged, which both reflects and furthers expectations about the (increasing) relevance of neuroscience to law (Pickersgill, 2011a). Neuroimages appear to be gaining traction within the courts – especially in the US, where the role of neurologic knowledge in law is expected by many to increase (e.g. Gurley and Marcus, 2008; Jones and Shen, 2012).

This is hardly a new phenomenon: representations of brains have been used to substantiate psychiatric arguments for almost as long as the technologies of their production have been expedient to use (Kulynych, 1996). It is not unexpected, either, given the longstanding recourse of law to technoscience in order to render transparent that which seems irresolvably opaque (Jasanoff, 2006). Yet, despite historical precedent the admissibility and reliability of neuroscientific evidence have been contested (see Patel et al., 2007). Although the question of whether ‘the use of neuroscientific evidence in the legal system will expand is an open and hotly debated question’ (Jones and Shen, 2012: 351), those individuals concerned with ‘neurolaw’ direct attention to the potential of neuroscience to impact on law – with some actively campaigning for the reshaping of legal systems and processes in light of neuroscientific insights. Such endeavours raise their own normative questions, given the ways in which anticipatory ethical discourse can play a key role in the development of sociotechnical futures, closing off certain avenues of technological development and governance regimes and legitimizing others (Hedgecoe and Martin, 2003). Perhaps with this in mind, ethicists have urged neuroscientists to ‘avoid inadvertently fueling [*sic*] misconceptions about the power and promise of neuroimaging’ (Kulynych, 2002: 355).

A related academic project that has been impelled by neuroscience is the discipline of ‘neuroethics’. The emergence of neuroethics has come at a time when ethicists have come to play an increasing role in the moral order of science, including attending to scientific and technological innovation and seeking to anticipate and analyse cultural and moral concerns before they concretize and individual or societal harm results. This reflexive governance of science (Braun et al., 2010) seeks to shape the future through practices of anticipation and consequent intervention (e.g. through professional codes of conduct, ethics training for scientists and recommendations by public bioethics bodies).

Anticipatory ethics is exemplified by neuroethics; this discipline engages actively in empirical and conceptual analyses that seek to identify and manage the emerging and potential consequences of neuroscience, answering the ‘host of new questions’ (Farah, 2012: 571) ethicists claim scientific research raises. At the same time, neuroethics aims to adjudicate existing concerns, such as enduring questions around addiction, brain death and informed consent, which are viewed as newly significant in light of the expanding scope of neuroscientific research and its perceived novelty (see, for example, Carter and Hall, 2011; Fuchs, 2006; Glannon, 2011; Illes et al., 2006). As other commentators have noted, this gives ‘a “hip” feel’ to neuroethics (Conrad and De Vries, 2011: 301); this has in turn enjoined critique of the discipline from both other bioethicists and social scientists, who have questioned the novel nature of the questions it asks and the degree to which it constructs neuroscience as innovative and transformative in order to provide support for the neuroethical enterprise (Conrad and De Vries, 2011; Brosnan, 2011; Martin et al., 2011; Rose, 2007).

In sum, then, we can see that the potential of and excitement associated with neuroscience animate new normative debates and areas of research (e.g. neuroethics) – reflecting a wider ‘preoccupation with interdisciplinarity’ (Barry et al., 2008: 21) within universities and beyond. At the same time, this assumed potentiality propels sociotechnical work between neuroscience and the professions (such as law), and ethical discussions about its consequences. By capitalizing on the perceived novel nature of neurologic knowledge, this work further substantiates and legitimates interdisciplinary praxis, endeavours to mutate expertise and claims to novelty. In what follows, I attend more closely to the implications for imagined, ascribed and experienced identities that are produced through these shifting configurations of authority and expertise.

## Biomedical identities and the cultures of care

As we have seen, neuroscience and its products are increasingly playing roles in (potentially and partially) reconfiguring ecologies of expertise in a range of contexts. In particular, this is through the production of hybrid knowledge and the absorption of neuroscientific concepts and data into sites that are distant from the laboratory. Universities are investing in and attracting sponsorship for initiatives and centres that seek to articulate neuroscience and diverse disciplines (like economics, music, philosophy and policy) with one another (Littlefield and Johnson, 2012; Schüll and Zaloom, 2011). These serve as sites within which different perspectives and methodologies might be brought into alignment and made to ‘stick’ (Molyneux-Hodgson and Meyer, 2009) together. In the process, they perform what, following Barry et al. (2008), we might call ‘ontological work’ (Pickersgill, 2011a).

As well as law and ethics, the promissory potential of neuroscience and the performance of ontological work is also apparent in (biomedicalized) debates around children and education. Here, neurologic narratives can be located within popular media and self-help books, as well as within policy documents and scientific literature on development. Within these texts, young people are constructed as what Choudhury et al. (2012) characterize as ‘neurological adolescents’, while the teaching profession is figured as a set of work practices that have much to benefit from neuroscience. Neuroscientific research is regarded by some of its practitioners as an endeavour that ‘is revealing, and will continue to reveal, a great deal about the developing brain’ (Blakemore et al., 2011: 6), providing ‘insights to guide innovative policies and practices’ (Shonkoff, 2011: 983). Parents, fuelled by desires that their offspring ‘exceed norms of behaviour and intellect’ (Nadesan, 2002: 413), can be active consumers of such discourses that present the brains of babies, children and young adults as plastic objects to be cared for, but also as biological constants that are determinative of behaviour and subjectivity. This exemplifies what Thornton (2011) has pointed to as a wider role of brain images in the public sphere, which act ‘to substantiate biological determinism’ whilst simultaneously supporting ‘claims that emphasize individual agency and responsibility’ (Thornton, 2011: 4; see also Pitts-Taylor, 2010). Such narratives implicitly – and sometimes explicitly – enjoin parents and teachers to take on board the lessons of neuroscience, (re)configuring their ascribed and perhaps enacted expertise in the process.

In line with widespread interest in what some have called ‘neuroeducation’ (e.g. Battro et al., 2008), the international publisher Elsevier has introduced a new title as part of their popular line of ‘Trends in …’ journals. This periodical, *Trends in Neuroscience and Education*, seeks to ‘bridge the gap between our increasing basic cognitive and neuroscience understanding of learning and the application of this knowledge in educational settings’ (Elsevier, 2012). Evoking narratives of scientific revolution, it is argued that:

Just as 200 years ago, medicine was little more than a mixture of bits of knowledge, fads and plain quackery without a basic grounding in a scientific understanding of the body, and just as in the middle of the nineteenth century, Hermann von Helmholtz, Ernst Wilhelm von Brücke, Emil Du Bois-Reymond and a few others got together and drew up a scheme for what medicine should be (i.e., applied natural science), we believe that this can be taken as a model for what should happen in the field of education. In many countries, education is merely the field of ideology, even though we know that how children learn is not a question of left or right political orientation. (Elsevier, 2012)

Yet, whilst the novelty and potential import of neuroscience is widely acknowledged, professionals at the coalface of practice, and even associations examining the role of neuroscientific research in (for instance) education, problematize and deconstruct the ascribed salience of the brain (Pickersgill et al., 2011). As the Royal Society put it in their report on ‘Neuroscience: Implications for Education and Lifelong Learning’:

There is great public interest in neuroscience, yet accessible high quality information is scarce. We urge caution in the rush to apply so-called brain-based methods, many of which do not yet have a sound basis in science. There are inspiring developments in basic science although practical applications are still some way off. (Royal Society, 2011: v)

This ‘great public interest’ appears most evidently instantiated through the media, where brain scans are readily apparent – especially in regard to discourses of health and illness. Images of brains, both normal and pathological, abound within magazines, books, newspapers and on television, with iridescent colours used to highlight regions that are ‘active’ or ‘hot’ and, it is assumed, thereby linked in some fundamental way to the subject of the commentary (Dumit, 2004). Images perform importance rhetorical work (Burri, 2012; Joyce, 2008); once produced, they can become central entities around which systems of authority and control orbit. In so doing, they at once contribute to the reification of some identities whilst reconfiguring others in sometimes surprising ways. The remarks and observations images are juxtaposed with in diverse media are often flagrantly promissory, extolling the power of the new brain sciences to reveal the secrets of the mind and cast fresh light on health, well-being and the very meaning of human nature. Nevertheless, these may be taken as entertaining, rather than profound, by audiences – or sometimes simply ignored (Pickersgill et al., 2011).

Particularly in regard to mental health, neuroscientific research and the images produced through it can also serve to bolster existing professional claims; psychiatric expertise is supported through the credibility attached to neuroscience in a range of domains. As suggested above, Rose perhaps overstates his case when he argues that whilst once ‘our desires, moods and discontents might previously have been mapped onto a psychological space, they are now mapped upon the body itself, or one particular organ of the body – the brain’ (Rose, 2007: 188). However, it seems clear that individuals diagnosed with mental disorder are often identified by a range of actors as being, in part, their brains, as a consequence of both the proliferation of neuroimages within popular media and the pharmacological treatments that are regularly prescribed to them to manage unruly subjectivities (Buchman et al., in press; Karp, 2006).

This use of psychopharmaceuticals is tightly bound up with the success of the third edition of the US *Diagnostic and Statistical Manual of Mental Disorders* (DSM-III, released 1980) (Healy, 2004). In particular, newly developed psychiatric drugs in the 1980s were trialled as specific treatments for the novel DSM-III diagnoses; following these, lavish symposia were sponsored through which the results of such studies could be disseminated. At the same time, DSM-III itself was heavily promoted internationally. The marketing efforts of both drug companies and the APA (American Psychiatric Association) were thus mutually beneficial: they powerfully reinforced both the legitimacy of the new diagnostic text and the categories it contained, and the efficacy of the compounds ostensibly developed to treat them. In recent years, the development of the Internet has enabled forms of direct-to-consumer advertising (Fox et al., 2006), further contributing to ways in which agents are subjectified as individuals living with mental ill-health.

Psychopharmaceuticals, and the neurobiological narratives that make sense of and are articulated through their promotion and consumption, are also ‘leaking’ (Lovell, 2006: 138) into spaces wherein they are employed to enhance normal functioning (rather than solely treat pathology). ADHD (attention deficit hyperactivity disorder) medication is a classic example of this, and wakefulness-promoting drugs are also important cases. The ‘off-label’ use of drugs in this way (e.g. by overworked students and business people) carries ‘the potential to reify and reinforce … dominant and hegemonic [cultural] narratives’ (Mamo and Fishman, 2001: 13). Chemical enhancement is indicative of a broader societal move away from viewing dependence on pharmaceuticals as a weakness, to instead considering their consumption as desirable or even essential in order to fully participate in society (Martin et al., 2011; Williams et al., 2008). The ‘duty to be well’ that Monica Greco (1993) has characterized as an animating theme within twentieth-century ‘Western’ society can now be understood as intimately entwined with what we might think of as a ‘duty to do well’ – and pharmaceuticals provide one of several means through which this might be achieved (though how many people will use them in this way remains an open question; Coveney, 2011).

At the same, though, criticism of the use of pharmaceuticals in this way is rife, not just for enhancement but for therapy as well (if a straightforward distinction between these can in fact be drawn). Lifestyle and psychosocial interventions for health and well-being continue to hold considerable traction within the UK and other ‘Western’ nations. Indeed, the implosion of selfhood into the brain has paradoxically acted to legitimize a range of applications of psychosocial knowledge, including intervention in families and children deemed ‘at risk’ of developing mental pathology and producing social disorder (Pickersgill, 2009; Walsh, 2011). The very fact that the brain is viewed as potentially shaped through ‘the environment’ underscores the continued salience of this ontological realm within public mental health. Indeed, neurobiological research may be reflexively rejected by psychiatrists and psychologists who read onto it assumptions of determinacy that undermine their therapeutic goals (Helén, 2011; Pickersgill, 2011b).

Further, in the UK at least, the ‘socio-medical organisation of psychiatric disorder’ (Prior, 1991: 403) seems to be shifting: developments in mental health policy today seek to prioritize psychological interventions, rather than drug therapies, with techniques such as cognitive behavioural therapy (CBT) increasingly lauded, funded and consumed. This is occurring in tandem with disinvestment treatments for mental illness by the pharmaceutical industry (Pickersgill, 2012b).^3^ One of the most significant biopolitical engines that has customarily been understood as driving the processes by which subjective experience comes to be mapped upon the brain thus could now to be powering down.^4^

## Discussion

As we have seen, neuroscience is frequently regarded as a powerful set of practices that can reveal important information about our brains and our selves. Such ascriptions of profundity reconfigure neuroscientists as not solely purveyors of fresh knowledge, but also co-producers (Jasanoff, 2004) of sociality as they enable and come to be embedded in new relationships with the health and other professions, patients and wider publics. With these developments come shifts in the flow of capital; neuroscience is a resource from which a new ‘promissory bioeconomy’ (Martin et al., 2008) is being formed. Neuroscience, through its construction as a novel epistemological practice, is clearly impacting on society in varied, subtle and sometimes far-reaching ways.

Alongside the perceived novelty of neuroscience sits its capacity to (re)legitimate older forms of knowing and acting. This raises questions regarding whether neuroscience may lend credibility to counter-modern (Beck, 1992) knowledge claims: for instance, to what extent might neuroscientific research (re)inscribe gender and ethnic differences onto biological sex? Alongside its modernist efforts to contribute to classical concerns of liberal democracies (health, education, justice), neuroscience therefore may also have implications for the exercise of new forms of power and control. This has not gone unnoticed by some academics and social groups: alongside celebrations of neuroscience sit passionate critiques labelling it as ‘reductionist’. The questions of whether new forms of discrimination may be engendered by neurologic conceptions of morality, deviance and sickness demand attention (cf. Kerr and Cunningham-Burley, 2000).

However, particular outcomes cannot be assumed. Further, it is not clear that neuroscientific knowledge is truly transformative, nor even that the celebratory, promissory and critical discourses associated with it are novel and unexpected. Rather, for over a century the scientific exploration of the brain has engendered both public and political interest, with many of the hopes invested in techniques like PET once instantiated within research involving technologies such as electroencephalography (EEG) (Borck, 2001, 2008; Hagner and Borck, 2001). Indeed, such tools produced particular models of the psyche (Borck, 2005) that can today be understood as the scaffolding upon which 21st century neurologic constructs have been assembled.

We must, then, be wary: not only of claims from neuroscientists and other actors about the potentiality of studies of the brain and the innovations they can and should engender, but also of highly theorized social scientific accounts that might over-play the novelty and import of neuroscience. As Webster (drawing on Barry, 2001) notes for genomics, it can be ‘difficult to identify novelty *in-itself*, since there is much evidence that shows how the *same* technoscience can be positioned and repositioned as old *and* new, depending on the networks and audiences it seeks to embrace or mobilise’ (Webster, 2005: 236, emphasis in original; see also Webster, 2002). Accordingly, it is problematic to view neuroscience as ‘intrinsically and necessarily transformative’ (Webster, 2005: 237), or, equally, as utterly lacking in social agency. Rather, the potential of neuroscientific research to (re)shape society is inextricably bound up in the social life of the brain: how, when and where is the neurological ‘consumed’, and in what settings and how does it gain traction and legitimacy?

As Vidal shows us, ‘brainhood predated reliable neuroscientific discoveries, and constituted a motivating factor of the research that, in turn, legitimized it’ (Vidal, 2009: 14). Today, social scientists themselves may play a role in the legitimization of neuroscience. Analysts are increasingly expected to play a ‘formative role’ (Barben et al., 2008: 983) in innovation through, for instance, collaboration and anticipatory governance (Molyneux-Hodgson and Meyer, 2009). This explicit positioning of social science within innovation policy has been broadly, but cautiously, welcomed by anthropologists, sociologists and STS scholars (Macnaghten et al., 2005), who today occupy ambivalent subject positions between ‘collaborator’, ‘contributor’ and ‘critic’ with and of scientific research and technological development (Calvert and Martin, 2009). Yet, such interdisciplinary working and engagement requires social scientists to be ‘involved explicitly in the construction of possible futures’ (Barben et al., 2008: 993); this future-orientated collaborative debate and practice is necessarily intertwined with discourses of novelty that legitimate interdisciplinarity. In turn, dialogue around the transformative potential of ‘new’ science, technology and medicine ‘plays an important role in informing and shaping social science research agendas’ (Hedgecoe and Martin, 2008: 817). This has created anxieties within social science communities regarding the extent to which researchers are serving to create further societal legitimacy for technoscientific practice (Burchell, 2009).

These wider debates resonate with the specific challenges indicated here regarding research into the place and role of neuroscience in society. Existing work has often drawn on Foucauldian insights around governmentality, emphasizing the kinds of self-making that encounters with the brain result in (Dumit, 2004; Nadesan, 2002; Pitts-Taylor, 2010; Rose, 2007; Thornton, 2011; Vrecko, 2006). This scholarship is rich and important, helping us to better understand the impacts of neuroscience on subjectivity. Yet, Foucault expressly argued for the possibilities of societal resistance to dominant discourses, which in the sociology of neuroscience have, to date, been less well documented and explored. Indeed, the very idea of a dominance/resistance binary presupposes widespread individual and institutional engagement with neuroscience and the neurological. Such an imaginary leaves insufficient room for elaborations of sociotechnical and subjective spaces wherein the brain is simply not present. As I have sought to describe here, the power of neuroscience is evident in a variety of social realms; yet, at the same time, it is also sometimes resisted or ignored. Moreover, the potency of neurologic technoscience is itself often activated by longstanding cultural tropes. This underscores a central question: how can social scientists engage with ‘new’ developments whilst recognizing that such novelty is itself socially constructed and may in fact be partly constituted *through* sociological engagement?

This article aims to articulate a problematic, rather than defend a solution. However, some further reflection on this question is important to set out here. In particular, sociological research must refrain from *assuming* novelty, even when case studies originate from or are propelled by a general understanding (held by the investigator, or evinced from them as apparent elsewhere) that some form of sociotechnical practice can be taken to be ‘novel’. In effect, I regard some kind of ‘methodological impartiality’ (cf. Bloor, 1991) as central to investigations into ‘novel’ technoscience. Perhaps of equal import, research must engage empirically (using a range of methods) with the matter of novelty: when, why and how is a particular realm of science or a kind of technological artefact deployed as ‘novel’ and to what epistemological or societal ends? Claims made through such research should likewise be reflexive about how partial they are: for instance, neuroscientists themselves might refuse to see their work as novel, whilst reporters often do, and wider publics may be ambivalent or agnostic. In bearing these issues in mind, sociologists might better chart the complex terrain from which science and technology emerge and within which they function, without inappropriately reifying the praxis under examination. However, this remains an incomplete response to the question detailed above: it demands further sociological engagement and debate.

## Conclusion

In this article, I have aimed to chart some of the key features of the current social life of the brain, whilst also documenting why, how and by whom the brain is (sometimes) taken to be important. The resonance of neuroscience in society is noteworthy to be sure; however, the brain itself might best be understood as being what I have elsewhere called an object of ‘mundane significance’ (Pickersgill et al., 2011). By this I mean that the brain – and, hence, studies of its function and structure – has a taken-for-granted salience that is synthesized through the considerable sociotechnical work that goes into figuring it as novel, yet simultaneously neuroscientific research is often distant from everyday professional and health practice. Considering the brain and the discourses and techniques that help to constitute it in this light acknowledges sociotechnical innovation but nevertheless remains mindful of the ways in which the ‘inventiveness’ (Barry, 2001) of neuroscience is itself socially produced. In so doing, cultural analyses of neuroscience might better distinguish themselves from biomedical and entrepreneurial discourses that likewise ascribe transformative effects to neurologic knowledge.

## Notes

1. Data collected include mental health policy documents, reports from bodies such as the Royal Society, journal articles in scientific and professional journals, (participant) observation at events pertaining to neuroscience, mental health and society (including clinical conferences and public engagement events), and interviews, focus groups and informal conversations with scientists, clinicians, service users and wider publics (including teachers, counsellors and members of the clergy).

2. The article does not provide a general schematic of the social, economic and epistemological drivers of neuroscience, which have been documented elsewhere (e.g. Beaulieu, 2001; Dumit, 2004; Littlefield and Johnson, 2012; Rose, 2007; Vidal, 2009).

3. For more on the economic, scientific and social aspects of disinvestment in psychopharmacology, see Chandler (in press).

4. Social scientists have shown extensively, though a wide range of richly ethnographic and detailed historical studies, how profoundly the pharmaceutical industry has shaped personal experience in the twentieth and twenty-first centuries; see Dumit (2012), Healy (2004), Jenkins (2009) and Petryna et al. (2006).
